# Reduced Mismatch Negativity is Associated with Increased Plasma Level of Glutamate in First-episode Psychosis

**DOI:** 10.1038/s41598-017-02267-1

**Published:** 2017-05-23

**Authors:** Tatsuya Nagai, Kenji Kirihara, Mariko Tada, Daisuke Koshiyama, Shinsuke Koike, Motomu Suga, Tsuyoshi Araki, Kenji Hashimoto, Kiyoto Kasai

**Affiliations:** 1 Department of Psychiatry, Kawamuro Memorial Hospital 71-Ko, Ohaza, Kitashinpo, Joetsu-shi, Niigata-ken, 943-0109 Japan; 2 Department of Neuropsychiatry, Graduate School of Medicine, University of Tokyo7-3-1, Hongo, Bunkyo-ku, Tokyo 113-8655 Japan; 3 Office for Counseling and Support, Division for Mental Health Support, The University of Tokyo 7-3-1, Hongo, Bunkyo-ku, Tokyo 113-8655 Japan; 4 Department of Rehabilitation, Graduate School of Medicine, University of Tokyo 7-3-1, Hongo, Bunkyo-ku, Tokyo 113-8655 Japan; 5 Department of Youth Mental Health, Graduate School of Medicine, University of Tokyo 7-3-1, Hongo, Bunkyo-ku, Tokyo 113-8655 Japan; 6 Division of Clinical Neuroscience, Chiba University Center for Forensic Mental Health 1-8-1, Inohana, Chuuoku-ku, Chiba-shi, Chiba-ken, 260-8670 Japan

## Abstract

Reduced amplitude of mismatch negativity (MMN) is one of the more promising biological markers of schizophrenia. This finding holds true in both early and chronic phases of the disorder, and is compatible with the glutamatergic dysfunction hypothesis. To further establish MMN as a biomarker of aberrant glutamatergic neurotransmission, an exploration for an association with blood levels of glutamatergic amino acids is an important next step. Despite a large body of work investigating MMN in schizophrenia, no previous studies have undertaken this endeavor. Nineteen patients with first-episode psychosis (FEP), 21 ultra-high risk individuals (UHR), and 16 healthy controls (HC) participated in the study. The MMNs in response to duration change (dMMN) and frequency change (fMMN) were measured. The fasting plasma levels of glutamate, glutamine, glycine, D-serine, and L-serine were measured. dMMN amplitudes were significantly reduced in FEP and UHR, compared to HC. The plasma levels of glutamate of FEP were significantly higher than those of HC. Higher plasma levels of glutamate were associated with smaller dMMN amplitudes in the FEP and HC groups. These findings are compatible with the hypothesis that MMN is a useful biological marker of aberrant glutamatergic neurotransmission in the early stages of schizophrenia.

## Introduction

Reduced amplitude of auditory mismatch negativity (MMN) is one of the more promising biological markers in schizophrenia^1–5^. MMN is an event-related potential (ERP) elicited when infrequent stimuli, deviant in physical features (e.g., duration, frequency [pitch]), occur in a sequence of repetitive auditory stimuli. MMN is thought to reflect auditory sensory memory or preattentive processing. N-methyl-D-aspartate (NMDA) receptor antagonists attenuate MMN amplitude in humans^6^ and in non-human primates^7, 8^; therefore, MMN is considered to be a biological indicator of glutamatergic neurotransmission. The reduced amplitude of MMN is conceptually compatible with the glutamatergic dysfunction hypothesis of schizophrenia^9–13^.

A number of studies have shown MMN amplitude attenuation, not only in chronic schizophrenia patients, but also in subjects in the early stages of psychosis^3, 14–20^. These findings suggest that MMN in response to duration deviant stimuli (dMMN) and MMN in response to frequency deviant stimuli (fMMN) may reflect different aspects of the pathophysiology in the early stages of psychosis^3^. Many studies have reported dMMN amplitude reduction in the early stages of psychosis. In individuals with ultra-high risk (UHR), dMMN amplitude reduction predicts the onset of psychosis^21^. On the other hand, few studies have reported fMMN amplitude reduction in the early stages of psychosis. In patients with first-episode schizophrenia, fMMN shows progressive reduction after the onset of psychosis^22^. While these findings indicate MMN amplitude reduction in early stages of psychosis as a group, MMN amplitude reduction is only present in a subset of individuals with early stages of psychosis in these studies. Because MMN is thought to reflect NMDA receptor function, the MMN amplitude attenuation in the subset may be due to the pathophysiology of NMDA receptor hypofunction. Therefore, MMN may serve as a biomarker to identify the subset that has psychosis based on NMDA receptor hypofunction.

To further establish MMN as a useful biological markers of aberrant glutamatergic neurotransmission, especially in the early stages of psychosis, the next step should be to investigate the relationship between MMN amplitude and blood level of molecules that influence NMDA receptor function, such as glutamate and D-serine. Peripheral glutamatergic amino acids have been investigated in patients with schizophrenia^23, 24^ and a meta-analysis showed that peripheral glutamate levels in schizophrenia patients were significantly higher than those in controls^24^. This is consistent with a prevalent model of hyperglutamatergic state in schizophrenia. This model is the so-called disinhibition mechanisms whereby NMDA antagonists block the NMDA-medicated drive on GABA interneurons that normally inhibit pyramidal cells. This leads to enhanced activity of pyramidal cells, which then results in increased glutamate release in adjacent cortical or afferent regions^25^.

However, to the authors’ knowledge, no studies have reported associations between MMN amplitude and peripheral glutamatergic amino acids in early stages of psychosis.

In this study, we measured MMN and the plasma levels of glutamatergic amino acids in subjects in the early stages of psychosis (UHR and first-episode psychosis [FEP]). We hypothesized the reduction of MMN amplitude, increased plasma level of glutamate, and an association between MMN amplitude and the plasma levels of glutamate in early stages of psychosis.

## Results

### Demographic and clinical variables

There were no significant differences in sex ratio, mean age, education, and premorbid IQ across the three groups. PANSS negative, general, or total were not significantly different between FEP and UHR. PANSS positive, GAF and antipsychotics dose were significantly different between FEP and UHR (Table 1).

**Table 1:** Demographic characteristics of study participants.

	FEP	UHR	HC	Statistics
N (sex ratio M/F)^a^	19 (14/5)	21 (12/9)	16 (11/5)	χ^2^ = 1.29, df = 2, p = 0.53
Mean age (years)^b^	23.5 (6.1)	21.3 (4.0)	22.3 (4.3)	χ^2^ = 0.91, p = 0.63
Education (years)^b^	13.9 (2.3)	13.4 (2.9)	14.2 (2.8)	χ^2^ = 0.35, p = 0.84
Premorbid IQ^c^	106 (9.2)	106 (9.3)	111 (6.9)	F_2,53_ = 1.53, p = 0.23
DUP (weeks)	19.5 (31.6)			
PANSS				
Positive	16.5 (5.1)	13.6 (3.3)		t_38_ = −2.16, p = **0**.04*
Negative	19.1 (6.9)	17.6 (5.3)		t_38_ = −0.46, p = 0.65
General	34.9 (2.1)	35.5 (6.9)		t_38_ = 0.52, p = 0.61
Total	71.0 (18.8)	66.7 (12.9)		t_38_ = −0.48, p = 0.64
GAF	39 (12.0)	48 (8.7)		t_38_ = 2.81, p = **0**.01*
Antipsychotic dose (mg/day)^d^	446 (280)	180 (345)		t_38_ = −2.65, p = **0**.01*

All values are shown as mean (standard deviation). Significant group differences are shown to the right. ^a^Chi-square test, ^b^Kruskal-Wallis test, ^c^one-way ANOVA. Otherwise, t-tests were used. P < 0.05 was considered significant. ^d^Chlorpromazine equivalent dose. Calculation for the mean antipsychotic dose include all patients including unmedicated ones. Abbreviations: FEP, first-episode psychosis; UHR, ultra-high risk; HC, healthy controls; IQ, intelligence quotient; PANSS, positive and negative symptom scale; GAF, global assessment of functioning; DUP, duration of untreated psychosis.

### MMN

The numbers of epochs for deviants in dMMN were 174 (±20) in HC, 156 (±31) in UHR, and 164 (±28) in FEP. One-way ANOVA revealed no significant difference among the three groups (F_2,53_ = 1.87, p = 0.17). The numbers of epochs for standards in dMMN were 1607 (±102) in HC, 1505 (±250) in UHR and 1496 (±257) in FEP. One-way ANOVA revealed no significant difference among the three groups (F_2,53_ = 1.34, p = 0.27).

**Figure 1:**
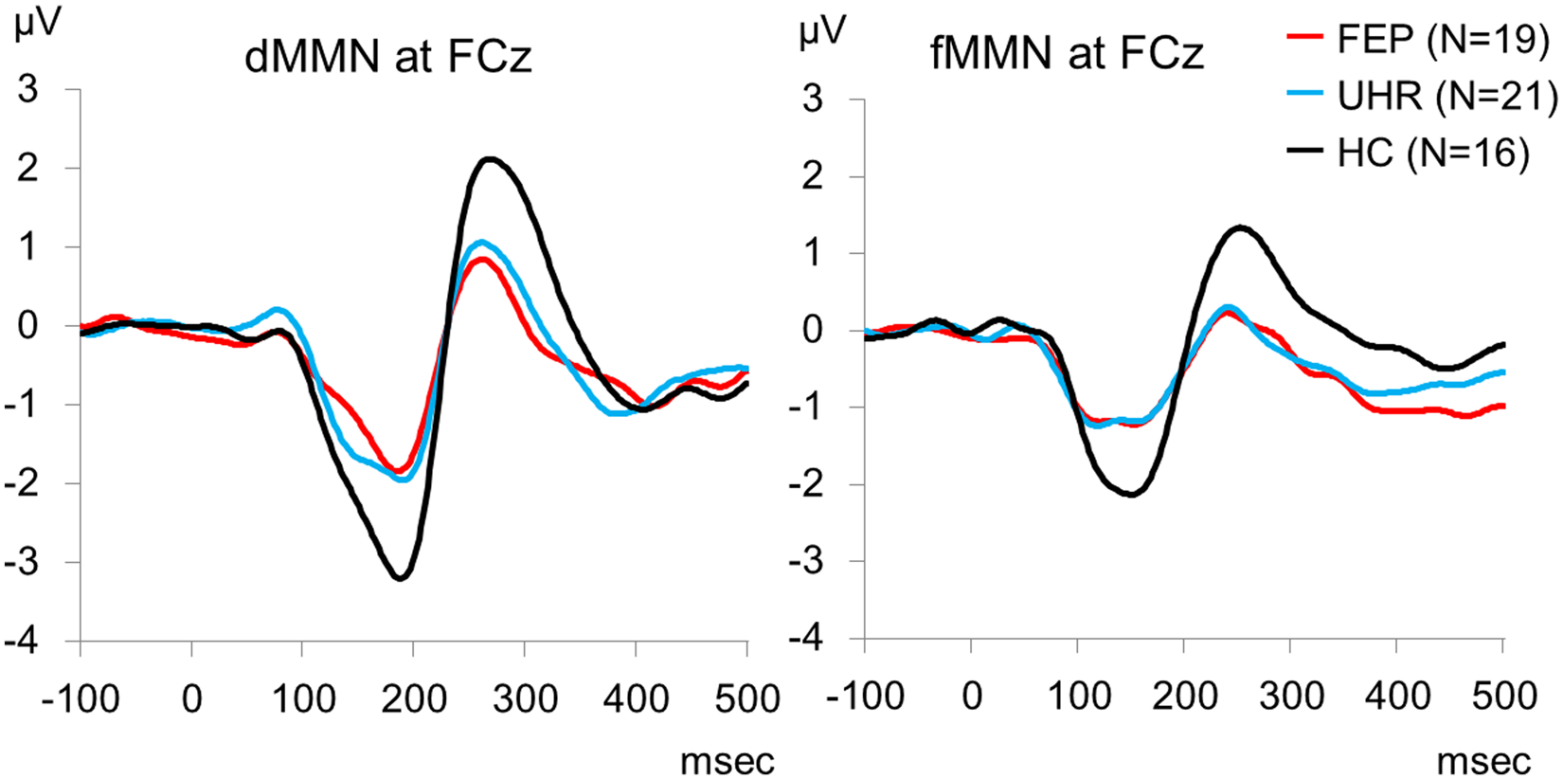
Grand average waveforms of duration mismatch negativity (dMMN) (left) and frequency mismatch negativity (fMMN) (right) at the electrode FCz in first-episode psychosis (FEP; red line), ultra-high risk (UHR; blue line), and healthy controls (HC; black line).

Figure 1 (left) represents the average waveform of dMMN at the electrode FCz. The data of dMMN amplitude of FEP, UHR, HC are all normally distributed according to Shapiro-Wilk test (p = 0.96, p = 0.09, p = 0.52, respectively). One-way ANOVA revealed significant group differences in dMMN amplitude (F_2,53_ = 6.41, p = 0.003, *η* ^2^ = 0.19). Post hoc tests with the Bonferroni correction showed significant differences in dMMN amplitude between FEP and HC (p = 0.004, Cohen’s d = 1.24), and between UHR and HC (p = 0.02, Cohen’s d = 0.88). There was no significant difference in dMMN amplitude between FEP and UHR (p = 1.00, Cohen’s d = 0.21).

The numbers of epochs for deviants in fMMN were 180 (±11) in HC, 156 (±27) in UHR and 167 (±33) in FEP. One-way ANOVA revealed significant difference among the three groups (F_2,53_ = 3.93, p = 0.026). Post hoc tests with the Bonferroni correction showed significant differences between UHR and HC (p = 0.021), but no significant differences between FEP and HC (p = 0.37) and between FEP and UHR (p = 0.64). There was no significant correlation between the number of epochs and fMMN amplitude in FEP (r_s_ = 0.26, p = 0.28), UHR (r_s_ = −0.03, p = 0.90) or HC (r_s_ = −0.49, p = 0.06). The numbers of epochs for standards in fMMN are 1595 (±118) in HC, 1522 (±207) in UHR and 1526 (±249) in FEP. One-way ANOVA revealed no significant difference among the three groups (F_2,53_ = 0.70, p = 0.50).

Figure 1 (right) represents the average waveform of fMMN at the electrode FCz. Among the data of fMMN amplitude of FEP, UHR, HC, those of UHR are not normally distributed according to Shapiro-Wilk test (p = 0.47, p = 0.00, p = 1.00, respectively). Kruskal-Wallis test showed significant difference in fMMN amplitude among the three groups. Howerver, Post hoc Mann-Whitney test with significance level p < 0.05/3 = 0.017 did not show any significant difference between HC and UHR (p = 0.025, r = 0.37), or between UHR and FEP (p = 0.728, r = 0.06), or between HC and FEP (p = 0.034, r = 0.36).

### The plasma levels of glutamatergic amino acids

**Figure 2:**
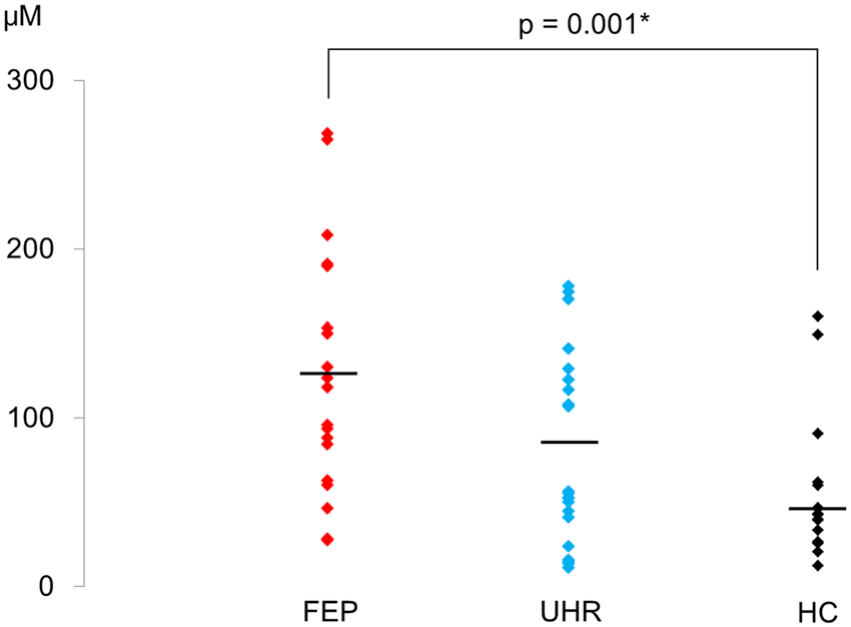
The plasma levels of glutamate in first-episode psychosis (FEP), ultra-high risk (UHR), and healthy controls (HC).

The plasma level of glutamate did not follow a normal distribution in FEP (p = 0.28), UHR (p = 0.03), and HC (p = 0.001) according to Shapiro-Wilk test. Kruskal-Wallis test showed significant difference in the plasma level of glutamate among the three groups (χ^2^ = 10.69, p = 0.005). Mann-Whitney tests with significance level p < 0.05/3 = 0.017 showed significant difference between HC and FEP (p = 0.001, r = 0.54) (Fig. 2). There was no significant difference between HC and UHR (p = 0.16, r = 0.24), or between UHR and FEP (p = 0.04, r = 0.32). The plasma levels of glutamine was normally distributed in FEP (p = 0.14), UHR (p = 0.36), and HC (p = 0.19) according to Shapiro-Wilk test. One-way ANOVA showed no significant difference in the plasma levels of glutamine among the three groups (F_2,53_ = 0.64, p = 0.53, *η* ^2^ = 0.02). The plasma levels of glycine were normally distributed in FEP (p = 0.52), UHR (p = 0.054), and HC (p = 0.18) according to Shapiro-Wilk test. One-way ANOVA showed no significant difference in the plasma levels of glycine among the three groups (F_2,53_ = 1.91, p = 0.16, *η* ^2^ = 0.07). The plasma levels of D-serine was normally distributed in FEP (p = 0.56), UHR (p = 0.34), and HC (p = 0.70) according to Shapiro-Wilk test. One-way ANOVA showed no significant difference in the plasma levels of D-serine among the three groups (F_2,53_ = 1.45, p = 0.24, *η* ^2^ = 0.05). The plasma levels of L-serine was normally distributed in FEP (p = 0.13), UHR (p = 0.10), and HC (p = 0.81) according to Shapiro-Wilk test. One-way ANOVA showed no significant difference in the plasma levels of L-serine among the three groups (F_2,53_ = 1.09, p = 0.34, *η* ^2^ = 0.04).

### The correlations between MMN amplitude and plasma levels of glutamatergic amino acids

**Figure 3:**
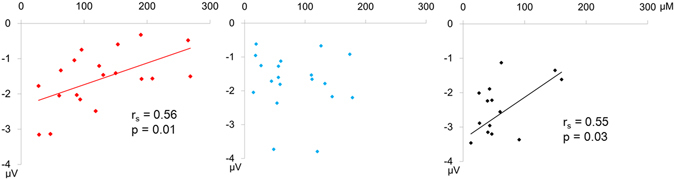
Correlations between duration mismatch negativity (dMMN) amplitude and the plasma level of glutamate at the electrode FCz in first-episode psychosis (FEP; left), ultra-high risk (UHR; middle), and healthy controls (HC; right).

The smaller amplitudes of dMMN were significantly associated with higher plasma levels of glutamate in FEP (r_s_ = 0.56, p = 0.01) and HC (r_s_ = 0.55, p = 0.03) (Fig. 3). UHR showed no significant correlations between them (r_s_ = 0.15, p = 0.52) (Fig. 3). We found no significant correlations between MMN amplitudes and the plasma levels of the other glutamatergic amino acids under investigation.

Although 18 patients with FEP and 9 individuals with UHR took antipsychotics, the antipsychotics dose was not significantly correlated with dMMN amplitude (FEP; r_s_ = 0.21, p = 0.39, UHR; r_s_ = 0.38, p = 0.09) or the plasma level of glutamate (FEP; r_s_ = 0.20, p = 0.41, UHR; r_s_ = −0.10, p = 0.67). In contrast, a higher antipsychotics dose was correlated with smaller fMMN amplitude in FEP (r_s_ = 0.51, p = 0.03).

### The correlations of clinical symptoms with MMN amplitude and plasma levels of glutamatergic amino acids

Neither MMN amplitude nor glutamate levels was significantly correlated with PANSS positive, negative, general or total in FEP or UHR (−0.27 < r_s_ < 0.36, p > 0.13).

## Discussion

The current study found significantly reduced dMMN amplitude and relatively preserved fMMN amplitudes in FEP and UHR. We also observed increased plasma levels of glutamate in FEP. Importantly, smaller dMMN amplitudes were associated with higher plasma levels of glutamate in FEP. These findings are compatible with the glutamatergic dysfunction hypothesis of schizophrenia, and confirm that dMMN is a useful biomarker of aberrant glutamatergic neurotransmission in the early stages of schizophrenia.

FEP and UHR showed significantly smaller dMMN amplitude and relatively intact fMMN amplitude compared to HC. These results are in line with previous studies^16, 26, 27^. Previous studies have shown that dMMN amplitude predicts the onset of psychosis in individuals with ultra-high risk^21^, and that fMMN amplitude may be associated with chronicity^1^.

The plasma level of glutamate of FEP was significantly higher than that of HC. The result is compatible with previous reports, which showed increased serum levels of glutamate in chronic schizophrenia patients^28, 29^. A couple of reports have attempted and failed to replicate these findings^30, 31^; however, a recent meta-analysis confirmed that the peripheral glutamate level of schizophrenia subjects was significantly higher than that of HC^24^. While these are findings pertain to chronic schizophrenia, we found increased glutamate levels in FEP. Since the present study used peripheral blood samples, it is uncertain whether the concentration of glutamate in the brain was also elevated. However, previous studies reported the peripheral glutamate levels were significantly correlated with cerebrospinal fluid (CSF) levels of glutamate^32, 33^. Furthermore, a recent meta-analysis reported that in schizophrenia, there were significant elevations in glutamate in the basal ganglia and no region showed a reduction in glutamate metabolites^34^. Therefore, the higher plasma glutamate concentration established in the present study might, at least in part, reflect an elevated glutamate level in the brain of FEP subjects.

Plasma levels of glutamine, glycine, D-serine, and L-serine showed no significant differences between FEP, UHR, and HC. Previous studies of peripheral levels of these glutamatergic amino acids in schizophrenia have yielded mixed findings: glutamine: significantly lower than HC^35^, comparable with HC^31^; glycine: higher^36^, lower^37^, comparable^38^; D-serine: higher^36^, lower^31, 38, 39^; L-serine: higher^40^, comparable^39^. Additionally, Hashimoto *et al*.^41^ reported that CSF glutamine levels were not different between schizophrenia subjects and HC. A recent meta-analysis showed the peripheral and CSF levels of glycine, D-serine, and L-serine were not significantly different between schizophrenia subjects and HC^23^, which corroborates our findings.

The most important finding of this study is that lower dMMN amplitudes were associated with higher plasma glutamate levels in FEP, although this inverse correlation did not appear to be specific to FEP; it was also observed in HC. There was no such association in UHR. To the authors’ knowledge, this is the first study that has reported the correlation between MMN amplitude and peripheral glutamate level in psychotic disorders. Although the molecular mechanism underlying the increased plasma level of glutamate in schizophrenia patients remains elusive, administration of NMDA receptor antagonists increased the concentration of glutamate in the prefrontal cortex in animals^42, 43^. Furthermore, administration of NMDA receptor antagonists attenuates MMN amplitude^6, 7, 44^. Therefore, hypofunction of NMDA receptors might cause increased plasma levels of glutamate and reduced MMN amplitude in FEP. The plasma level of glycine, D-serine and L-serine neither decrease nor correlate with MMN amplitude in FEP or UHR. These results suggest that NMDA hypofunction in schizophrenia may not due to the decrease of NMDA receptor co-agonists. The plasma level of glutamine also showed no significant correlation with MMN amplitude in FEP or UHR. Because glutamine has no agonistic activity at NMDA receptors, the plasma level of glutamine may not reflect NMDA receptor function.

Although dMMN amplitude was lower and the plasma level of glutamate was higher in FEP compared to HC, not all patients with FEP had reduced amplitude of dMMN and increased level of glutamate (Fig. 3). These results suggest that not all patients with FEP but a subset of FEP may have hypofunction of NMDA receptors. Therefore, dMMN and the plasma level of glutamate may serve as biomarkers to differentiate individuals with hypofunction of NMDA receptors from individuals without hypofunction of NMDA receptors in patients with FEP. According to a recent meta-analysis, glutamate positive modulators may not be effective in reversing overall cognitive impairments in patients with schizophrenia^45^. However, glutamate positive modulators may be effective only in a subset of the patients who has NMDA receptor hypofunction. The plasma level of glutamate and dMMN may be useful for identifying this subset. Future clinical trials using dMMN and the plasma level of glutamate as biomarkers will be useful for developing new treatments that modulate NMDA receptor function. These stratified clinical trials based on biomarkers will lead to precision medicine for early stages of psychosis that is the objective of the Research Domain Criteria (RDoC)^46^.

We investigated association between MMN and glutamatergic amino acids in psychosis based on glutamatergic hypothesis of schizophrenia. However, previous studies reported the reduced amplitude of MMN in other mental disorders such as bipolar disorder^47^. In addition, bipolar disorder also shows aberrant glutamate transmission^48^. Therefore, the association between reduced amplitude of MMN and increased plasma level of glutamate may not be specific to psychosis. However, future studies with a sample including various mental disorders will be needed to investigate the specificity of the association between reduced amplitude of MMN and increased plasma level of glutamate.

We have several limitations in this study. First, although the antipsychotics dose was not significantly correlated with dMMN amplitude, nor with the plasma level of glutamate, correlational analyses may be insufficient to clarify potential medication effects. Further studies with drug-naïve participants would be necessary to investigate whether the association between dMMN and plasma level of glutamate reflect NMDA pathology of schizophrenia or potential medication effects. Second, since the present study is a cross-sectional one, the association between dMMN amplitude and plasma level of glutamate is correlative. A longitudinal study, currently underway in our laboratory, will clarify the association during the early stages of schizophrenia. Third, a sample size was small in this study. Further studies with a large sample size will be needed to confirm the correlation between MMN and the plasma level of glutamate.

In conclusion, we found reduced dMMN amplitude and increased plasma level of glutamate in FEP, and a significant association between the two indices. These findings suggest that dMMN and plasma glutamate levels may be useful biological markers for altered glutamatergic neurotransmission in the early stages of schizophrenia. If replicable in larger-scale studies, they may represent promising candidates for biomarkers to be utilized in real-world clinical settings, which has, thus far, been a great challenge in psychiatry.

## Methods

### Participants

Nineteen patients with FEP, 21 individuals with UHR and 16 healthy controls (HC) participated in this study (Table 1). MMNs from a subset of these participants were previously published^16^. Thirteen FEP, 7 UHR and 10 HC were included in the previous study who agreed to blood drawing and the examination of the plasma level of amino acids, and the rest were newly recruited for the present study. All participants with FEP or UHR were recruited from The University of Tokyo Hospital. All individuals with UHR, and most patients with FEP, were help-seeking individuals registered at the University of Tokyo Hospital outpatient unit specialized for early intervention. HC were recruited through advertisements placed at several universities near The University of Tokyo. This study was conducted as a part of our large-scale, multimodal, neuroimaging and psychophysiological studies in the early stages of psychosis (Integrative Neuroimaging Studies for Schizophrenia Targeting Early Intervention and Prevention; IN-STEP). Koike *et al*.^49^ reported the detail of the recruitment protocol. All subjects gave written informed consent to the Research Ethics Committee of the Faculty of Medicine, The University of Tokyo (approval Nos 629-8, 2094-7, 2226-4) following a complete explanation of this study, and in accordance with the Declaration of Helsinki. Inclusion and exclusion criteria for each group are shown in Table 2. We used the Structured Interview for Prodromal Symptoms (SIPS) as the UHR criteria, which consists of 3 criteria: attenuated psychotic symptoms (APS), brief intermittent psychotic symptoms (BIPS), or genetic risk and deterioration (GRD)^50, 51^. APS correspond to individuals who exhibited onset or worsened subthreshold psychotic symptoms within 12 months but not psychotic severity. BIPS correspond to individuals who had psychotic symptoms within 3 months but with a limited duration and frequency such that they were not at all or only slightly influenced by their symptoms and did not meet the psychotic episode criteria according to the DSM-IV criteria^52^. GRD corresponds to individuals whose functioning had deteriorated in the previous 12 months as defined by a 30% or more decrease in the GAF score^52^ as well as those who also had one or more first-degree relatives diagnosed with psychosis and/or schizotypal personality disorder according to the DSM-IV criteria.

**Table 2:** Inclusion and exclusion criteria.

First-episode psychosis (FEP) group
• Diagnosis of FEP using the Structured Interview for Prodromal Symptoms (SIPS)^50, 51^
• Age 15–40 years
• No history of antipsychotic drug treatment for more than 16 cumulative weeks
• Continuous psychotic symptoms within the past 60 months
Ultra-high risk for psychosis group
• Diagnosis of UHR using SIPS
• Age 15–30 years
• No history of antipsychotic drug treatment for more than 16 cumulative weeks
Healthy control group
• Age 15–40 years
• No personal history of psychiatric disease or a family history of axis I disorders in their first-degree relatives
Exclusion criteria for all groups
• Neurological illness
• Traumatic brain injury with any known cognitive consequences of loss of consciousness for more than 5 min
• History of electroconvulsive therapy
• Low premorbid IQ (below 70)
• Previous alcohol/substance abuse or addiction

Abbreviations: IQ, intelligence quotient.

Audiometer testing was used to ensure that all participants had normal hearing in both ears and could detect 30-dB sound pressure level tone at 1000 Hz and 40-dB at 4000 Hz. Eighteen FEP and 9 UHR subjects were prescribed second-generation antipsychotic medications at the time of testing. No other antipsychotic was prescribed. All patients with FEP were diagnosed by experienced psychiatrists according to the DSM-IV criteria. Out of 19 FEP patients, 17 patients had first-episode schizophrenia, and 2 patients had schizophreniform disorder. Estimated premorbid intelligence quotient (IQ) was assessed with the Japanese version of the National Adult Reading Test^53, 54^ FEP and UHR participants were assessed with regards to their functioning and symptoms, using the Global Assessment of Functioning (GAF)^52^ and the Positive and Negative Syndrome Scale (PANSS)^55^. These clinical ratings were measured by experienced psychiatrists (T.N., M.T., and D.K.). To control the quality of data assessment, we used video interviews to ensure the scoring of the clinical ratings and calculated the inter-rater reliability of the clinical ratings (Cronbach alpha = 0.86)^49^.

### Measurement of the plasma levels of amino acids

We measured plasma levels of the peripheral glutamatergic amino acids glutamate, glutamine, glycine, D-serine, and L-serine. Measurement of the plasma levels of glutamate, glutamine, and glycine was carried out using a column-switching high performance liquid chromatography (HPLC) system (Shimadzu Corporation, Kyoto, Japan) with fluorescence detection, as has been previously reported^41^. Measurement of D-serine and L-serine was carried out using a HPLC system, as has been previously reported^39, 56^. In order to minimize the effect of amino acids originating from food sources, we obtained fastening (>3 h without any meals and/or nutritious drinks) blood samples, as has been previously reported^57^.

### Stimuli and Procedure

For duration MMN (dMMN), a two-tone auditory oddball paradigm with 2000 stimuli was used. Ninety percent of the stimuli were standard tones (1000 Hz, 50 ms) and 10% of the stimuli were deviant tones (1000 Hz, 100 ms). For frequency MMN (fMMN), another two-tone auditory oddball paradigm with 2000 stimuli was used. Ninety percent of the stimuli were standard tones (1000 Hz, 50 ms) and 10% of the stimuli were deviant tones (1200 Hz, 50 ms). All the stimuli were of 80 dB sound pressure level and 1 ms rise/fall time. Stimulus onset asynchrony was 500 ms. These oddball paradigms were counter-balanced. Tones were presented binaurally through earphones while participants watched a silent cartoon.

### Electroencephalography recording and analyses

Electroencephalography (EEG) data were acquired with a 64-channel Geodesic EEG System (Electrical Geodesics Inc., Eugene, OR). Electrodes were referenced to the vertex, and impedances were kept below 50 kΩ. The sampling rate was 500 Hz. The analog filter bandpass was set at 0.1–100 Hz. EEGLAB^58^ was used for EEG processing and data analysis. The continuous EEG data were re-referenced to an average reference, digitally filtered at 0.1–20 Hz, and segmented from −100 to 500 ms relative to the stimulus onset. The mean of the pre-stimulus baseline was subtracted for baseline correction. Independent component analysis was used for eye blink correction. Epochs exceeding ±100 μV at any electrode were rejected. After averaging across trials, the ERP waveform in response to standard stimuli was subtracted from the ERP waveform in response to deviant stimuli.

For MMN analysis, a front-central electrode (FCz) was selected (the largest amplitude was obtained with this FCz). The dMMN amplitude was measured as the mean voltage from 135 to 205 ms post stimuli, in accordance with previous studies^16, 59, 60^. The fMMN amplitude was measured as the mean voltage from 100 to 200 ms, in accordance with previous studies^16, 61, 62^.

### Statistical Analyses

We performed Shapiro-Wilk tests to examine whether each variable follows a normal distribution. When the variable followed a normal distribution, we employed independent t-test for comparison between 2 groups and one-way analysis of variance (ANOVA) for comparison among 3 groups. When the variable did not follow a normal distribution, we employed Mann-Whitney test for comparison between 2 groups and Kruskal-Wallis test for comparison among 3 groups. We employed a χ^2^ test for sex ratio.

When we found significant difference in one-way ANOVA, Post hoc tests with the Bonferroni correction were performed to examine group difference. When we found significant difference in Kruskal-Wallis test, Mann-Whitney tests with the Bonferroni correction were performed to examine group differences.

We reported effect sizes using Cohen’s d for variables with normal distribution, r for variables with non-normal distribution, and η^2^ for ANOVA.

To assess the association of MMN amplitude with the plasma levels of glutamatergic amino acids, Spearman correlation coefficients were calculated. The significance level was set at p < 0.05.
